# Genome-wide association studies and genetic architecture of common human diseases

**DOI:** 10.1186/1753-6561-5-S4-S16

**Published:** 2011-06-03

**Authors:** Grant W Montgomery

**Affiliations:** 1 Molecular Epidemiology Laboratory, Queensland Institute of Medical Research, Brisbane, Australia

## Abstract

Genome-wide association scans provide the first successful method to identify genetic variation contributing to risk for common complex disease. Progress in identifying genes associated with melanoma show complex relationships between genes for pigmentation and the development of melanoma. Novel risk loci account for only a small fraction of the genetic variation contributing to this and many other diseases. Large meta-analyses find additional variants, but there is current debate about the contribution of common polymorphisms, rare polymorphisms or mutations to disease risk.

## Background

Genome-wide association (GWA) methods have made great progress in the last few years mapping genetic variants contributing to risk for many common complex diseases [1-3]. These methods developed from spectacular advances in genotyping technology, greater understanding of the structure of common variation in the human genome, and continued advances in computing power and software tools. Human populations have 10 to 15 million common single nucleotide polymorphisms (SNPs). Analysis of patterns of SNP variation in the human genome [4] demonstrate that a representative set of 500,000 to 1,000,000 “tagging” SNPs can sample most common variation. Current commercial SNP chips can now screen genome-wide “tagging” SNPs in a single experiment and provide an effective approach to search for genetic variants contributing to the aetiology of complex diseases.

## Results and discussion

### Pigmentation and melanoma

One example of mapping genes for related phenotypes is the field of pigmentation and melanoma. Melanomas ([MIM 155600]) are malignant tumours of melanocytes and susceptibility is influenced by complex relationships between genetic and environmental factors [5,6]. Risk factors include skin pigmentation (skin colour and tanning response or phototype) and the numbers of acquired melanocytic nevi or moles on the skin [5,6]. These intermediate phenotypes interact with the key environmental factor, exposure to ultraviolet light.

The incidence of malignant melanoma in Caucasian populations has increased substantially over the last 20 years [6] probably as a result of long-term changes in sunexposure.

A number of GWA studies have been conducted recently to improve our understanding of the genetics of pigmentation and contributions to melanoma risk [7-13]. Most studies have been conducted in Caucasian populations and it is important to note that variants in several pigmentation genes show significant differences in frequency, and are responsible for differences in pigmentation between ethnic groups. Therefore, care must be taken to avoid problems of population stratification in the design and interpretation of studies on pigmentation and melanoma risk.

GWA studies have identified both known and novel pigmentation genes and results have been replicated and extended by further studies including a recent meta-analysis [14]. Genes controlling pigmentation and tanning response include *ASIP* [MIM 600201], *IRF4* [MIM 601900], *KTLG* [], *MC1R* [MIM 155555], *OCA2* [MIM 611409], *SLC45A2* [MIM 606202], *SLC24A5* [MIM 609802], *SLC24A4* [MIM 609840], *TYR* [MIM 606033], *TYRP1* [MIM 115501]. Variants in or near genes *ASIP*, *KTLG*, *MC1R*, *OCA2*, *IRF4*, *SLC24A4*, *SLC45A2*, also affect sun sensitivity and/or melanoma risk [14].

In some cases the variants are located within coding regions and have functional consequences [15]. However, most variants will not be causal and the association is a consequence of linkage disequilibrium between the marker and the causal variant(s). For example, *OCA2* has long been implicated as an important gene affecting blue/brown eye color. Genetic analysis of SNPs in the gene *HERC2* which lies upstream from *OCA2* (in the direction of transcription for *OCA2*) identified a single SNP rs12913832 in intron 86 of *HERC2* accounting for most of the variation in blue/brown eye colour [16,17]. This SNP lies in the centre of a short highly conserved sequence which forms a consensus binding site for the helicase-like transcription factor (*HLTF*) and is likely to control constitutive expression of *OCA2*[17]. This and other examples demonstrate that causal variants may lie in flanking genes. In regions of association where multiple genes may influence risk of melanoma or other diseases, additional studies will be necessary to determine the likely causal variant(s) and define the gene or genes responsible for the phenotypic effect.

### Moles and melanoma risk

The most important risk factor for melanoma is the number of acquired moles [5]. Depending on the population under study, the risk for melanoma increases by 2%–4% for each additional mole counted and individuals in the top 10% of the mole count distribution have a 5- to 10- fold higher risk. Sun exposure interacts with both pigmentation and development of moles, but there is evidence for “divergent pathways” for the roles of UV radiation exposure and high mole count in development of melanoma (Whiteman 2003). Individuals with lower melanocyte proliferation and few moles develop melanoma on body sites with high cumulative UV radiation exposure while those with high melanocyte proliferation develop melanoma on body sites with lower UV radiation exposure and more moles. Total mole count has a high heritability of ~70% [18,19] and about half the genetic variance for mole count can be attributed to a locus in the region of *CDKN2A* (MIM 600160) on chromosome 9 [9,18,19]. High-penetrance coding mutations in *CDKN2A* are reported in families with multiple melanoma cases and these families also carry greater numbers of nevi. However, the *CDKN2A* variants exist at population frequencies of less than 0.1%, and so explain no more than 1%–2% of melanomas in the general population. A GWA study of mole count identified common SNPs in *MTAP* (MIM 156540) associated both with mole count and melanoma risk [9]. *MTAP* is located adjacent to *CDKN2A* on chromosome 9p and it is not known whether the risk alleles in the 5’ region of *MTAP* act through direct effects on *MTAP* or through effects on *CDKN2A*. The same study also demonstrated association with mole count and melanoma risk for a second locus on chromosome 22q13. The strongest association signal was for a SNP in the second intron of *PLA2G6*, a gene belonging to the phospholipase A2 (PLA2) super family of genes [9].

The gene IRF4 is also associated with mole count, but shows a strong gene x age interaction [20]. The T allele for rs12203592 located in intron 4 of IRF4 was associated with high mole counts and high freckling scores in adolescents, but with low mole counts and high freckling scores in adults. The C allele (associated with higher mole count in adults) was also associated with melanoma risk, most significantly with melanoma on the trunk. The gene x age interaction could easily have been missed in a single sample combining individuals of different age groups.

GWA studies have made good progress in identifying genes contributing to variation in pigmentation, mole development and melanoma risk. It is estimated that variants so far identified for genes influencing skin, eye and hair colour and tanning response account for about half of the melanoma risk due to pigmentation [21]. In contrast, only 2% of variation has been explained for non-pigmentation factors associated with melanoma risk including mole count. However, many of these effects are likely to act through melanocytes and *CDKN2A*, implicated directly in mole development and melanoma risk, has associated pigmentation effects in chickens [22]. Missense mutations in the coding region of *CDKN2A* are responsible for sex-linked barring, a common plumage colour characterized by black and white barred feathers. These studies illustrate the complex relationships between genes and environment in pigmentation and the development of melanoma.

### Effect size and missing variation

GWA methods have been very successful in identifying genes and variants associated with common diseases and these discoveries have provided new insights into the biology of many diseases. However, the effect sizes for individual variants are generally small with odds ratios for the risk alleles in the range of ~1.1 to ~1.5. Pigmentation variation has been under strong selection and there are large effects reported for some individual variants. In contrast, the effects of variants associated with melanoma risk are more modest and typical of effect sizes for variants associated with most common diseases. Collectively, known variants for individual diseases only account for a small fraction of the familial risk or heritability [2,3]. One approach to this problem has been to combine results from many studies and conduct meta-analysis of results with sample sizes of over 100,000 individuals. This approach is only possible for diseases or phenotypes where many samples have been collected with the same or similar disease definitions. Some recent examples include the analysis of smoking behavior in 74,000 individuals [23], and serum lipids in >100,000 individuals [24]. These large studies have greatly increased power and each identify many novel associated variants. However, in most cases the combined results still only explain a small proportion of the genetic variation. There has been much debate about the source of the other “missing” variation. The two main possibilities are that most causal variants are not tagged well by SNPs on commercial chips (e.g. because they occur at lower frequency or are in areas of the genome for which it is difficult to develop SNP assays), or genetic contributions to disease risk are due to many variants with odds ratios so small that they do not reach formal statistical significance despite large GWA studies. Current commercial SNP chips generally target common variants. Ability to tag causal variants depends on linkage disequilibrium, in turn influenced by differences in allele frequency between markers and low frequency or rare variants will not be well “tagged” by SNP markers on many current chips.

### Contribution of rare variants

Contribution to disease risk in the population is a function of allele frequency and also of effect size for the risk allele. Rare disease associated variants not tagged by current chips can only be the source of missing heritability if the risk alleles have large effects. Re-sequencing of genomic regions uncovers new variation and there are a number of examples where rare variants contribute to risk for common traits. Rare variants in CDKN2A discussed above contribute to melanoma in high risk families, but explain little of the population prevalence for this disease. *GDF9* is expressed in human oocytes and plays important roles in growth and selection of ovarian follicles. A search for *GDF9* variants in mothers of spontaneous DZ twins identified three novel deletions and four mis-sense alterations [25,26]. Taken together, the frequency GDF9 variants were significantly higher in mothers of DZ twins compared with controls [25,26]. However, the frequency of the variants is low (less than 4% for all variants) and the contribution of these variants to the overall incidence of twinning is small. Resequencing a candidate gene for type 1 diabetes detected new variants at ~1% frequency that in total contributed more to variation in risk in the population than a single common variant in the same gene detected by a previous GWA study [27]. Recently, GWA identified common variants APOA5, GCKR, LPL and APOB associated with hypertriglyceridemia (HTG, [28]). Resequencing of these genes revealed a significantly higher burden of rare missense or nonsense variants in individuals with HTG, compared to controls corresponding to a carrier frequency of 28.1% of affected individuals and 15.3% of controls. Common genetic variants in seven HTG-associated loci explained ~20% of total variation in HTG diagnosis, and the rare genetic variants in four HTG-associated loci explained ~1% of variation. Therefore, both rare and common variants in the same genes can influence disease risk. Based on current examples, the contributions from rare or low frequency variants are similar to common variants and much variation in genetic contributions to disease risk is still “missing”.

### Common variants of small effect explain missing heritability

Most GWA studies have examined evidence for association SNP by SNP. An alternative approach is to analyse data for all SNPs together to estimate the proportion of trait variance accounted for by all common variation “tagged” by the SNPs on current commercial SNP chips. This is possible because the distant genetic relatedness of individuals can be estimated from dense SNP data. Once the degree of relatedness is established, it can be compared to phenotypic similarity between the individuals. This method was developed and used to estimate the genetic contribution to variation in height independent of the usual assumptions required to estimate heritability using family data [29]. Using this approach, the percentage of phenotypic variation explained by common SNPs was 45%.

This is less than the 80% of phenotypic variance due to additive genetic effects based on the estimated heritability. However, the SNPs sampled on the arrays may not be in complete LD with the causal variants and this might influence the results. If the estimate is corrected first for the sampling error from using a finite number of SNPs with genotype data, the corrected estimate for variance explained by causal variants is 54% (assuming the same structure of linkage disequilibrium between causal variants and common SNPs sampled on the arrays). In addition, if the causal variants tend to have lower minor allele frequencies than SNPs on the arrays, we would expect lower LD between genotyped SNPs and causal variants [29]. When this is taken into account, the estimated contribution of phenotypic variation explained was 84%. The standard error for this estimate is large and it does not prove that causal variants do have lower allele frequencies than tagging SNPs used on the chips. However, if this were the case, most of the phenotypic variation in height due to additive genetic effects could be explained by many common variants with small effects [29]. Whether this applies only to human height or more generally remains to be seen.

## Conclusions

GWA studies have identified a large number of variants associated with a range of human traits and common diseases. However, the sizes of effects on disease risk are typically small. Combining results across many studies increases the power to detect risk variants and resequencing is uncovering rare variants with modest contributions to a number of diseases. The emerging view from all these studies is a spectrum of many variants with small effects explaining genetic contributions to disease risk.

The discoveries provide new insights into the biology of many diseases with a number of variants located in genes that contribute to biological pathways not previously considered to be involved in disease, or located in regions that do not contain known protein-coding genes. Some examples like the effects of IRF4 on mole count show interactions that would reduce estimated effects size from large combined studies. Therefore one important outcome of GWA studies will be to use knowledge gained to evaluate genetic contributions to disease sub-classes, disease heterogeneity and co-morbidity for different diseases. The next challenge is how to translate these discoveries into better diagnostic practices, preventions and treatments.

## Competing interests

The authors declare that they have no competing interests.
